# A Simple Method to Decode the Complete 18-5.8-28S rRNA Repeated Units of Green Algae by Genome Skimming

**DOI:** 10.3390/ijms18112341

**Published:** 2017-11-06

**Authors:** Geng-Ming Lin, Yu-Heng Lai, Gilbert Audira, Chung-Der Hsiao

**Affiliations:** 1 Laboratory of Marine Biology and Ecology, Third Institute of Oceanography, State Oceanic Administration, Xiamen 361005, China; lingengming@tio.org.cn; 2 Department of Chemistry, Chinese Culture University, Taipei 11114, Taiwan; lyh21@ulive.pccu.edu.tw; 3 Department of Bioscience Technology, Chung Yuan Christian University, Chung-Li 32023, Taiwan; gilbertaudira@yahoo.com; 4 Center for Biomedical Technology, Chung Yuan Christian University, Chung-Li 32023, Taiwan; 5 Center for Nanotechnology, Chung Yuan Christian University, Chung-Li 32023, Taiwan

**Keywords:** green algae, rRNA repeated unit, phylogeny, genome skimming

## Abstract

Green algae, *Chlorella ellipsoidea*, *Haematococcus pluvialis* and *Aegagropila linnaei* (Phylum Chlorophyta) were simultaneously decoded by a genomic skimming approach within 18-5.8-28S rRNA region. Whole genomic DNAs were isolated from green algae and directly subjected to low coverage genome skimming sequencing. After de novo assembly and mapping, the size of complete 18-5.8-28S rRNA repeated units for three green algae were ranged from 5785 to 6028 bp, which showed high nucleotide diversity (π is around 0.5–0.6) within ITS1 and ITS2 (Internal Transcribed Spacer) regions. Previously, the evolutional diversity of algae has been difficult to decode due to the inability design universal primers that amplify specific marker genes across diverse algal species. In this study, our method provided a rapid and universal approach to decode the 18-5.8-28S rRNA repeat unit in three green algal species. In addition, the completely sequenced 18-5.8-28S rRNA repeated units provided a solid nuclear marker for phylogenetic and evolutionary analysis for green algae for the first time.

Green algae, a big group which contains at least 7000 species, has been found in wide range habitats from freshwater to sea water [1]. Similar to land plants, green algae contain chlorophyll *a* and chlorophyll *b* and store food as starch in plastids [2]. In the ecosystem, green algae play a role as primary photosynthetic eukaryotic organisms. The green algae have become powerful producers and providers of various natural substances, which may constitute the primary natural source, such as minerals, vitamins, nutrients, and fatty acids, as well as carotenoid pigments that include carotenes, xanthophylls, zeaxanthin, and lutein [3,4,5]. Currently, it is feasible to produce some carotenoids commercially through aquaculture [6]. In addition, because of their rapid growth and high oil content, some green algae also have been considered as a promising alternative feedstock for biodiesel production [7,8]. However, the challenge of developing green algae as a permanent fuel source will be to operate industries sustainably and compete with existing energy options with its costly investment [9].

In spite of their many unifying features, green algae exhibit remarkable in morphology and ecology reflecting their evolutionary diversification. Recently, according to cladistic classification and molecular analysis, the monophylogenic of green algae origin is still arguable [10,11,12,13], which referred that more approaches are required to validate phylogeny of green algae at molecular level by using different markers. The rapid increase in genomic data from a wide range of green algae has high potential to resolve large-scale green algal relationships. Furthermore, green algal genomes are important sources of information for the evolutionary origins of plant traits due to their evolutionary relationship to land plants [14,15]. Therefore, in this study, we demonstrated a genome skimming method to deduce the complete 18-5.8-28S rRNA repeated sequence (as a nuclear marker), which used as a molecular tool to reveal the relationships between *Haemotococcus pluvalis*, *Chlorella ellipsoidea* and *Aegagropila linnaei* to discuss the relationship between the freshwater and marine algae. 

Genome skimming is an approach which reconstructs whole genome shotgun libraries faster and easily. This technique involves filtering millions of shotgun next generation sequencing (NGS) reads to find the few reads associated with particular DNA regions of interest [16], which is 18-5.8-28S rRNA repeated sequence for this case. Internal transcribed spacers (ITS) are sequences located in eukaryotic rRNA genes between the 18S and 5.8S rRNA coding regions (ITS1) and between the 5.8S and 28S rRNA coding regions (ITS2). The ITS is a non-coding region with high interspecific variability allowing differentiation of species within a genus, but low intra-specific variability preventing the separation of individuals or strains within the same species. These spacer sequences are present in all known nuclear rRNA genes of eukaryotes and have a high evolution rate. Previous restriction site variation studies in the ribosomal DNA (rDNA) have shown that the spacer regions are variable while coding regions are conserved. ITS are useful for phylogenetic analysis among related species and among populations within a species [17]. Combined with genome skimming, we implemented a rapid and cost-effective strategy for generating phylogenetically informative genomic data [18,19].

*Haematococcus pluvialis*, an unicellular green algae, is a promising microorganism that now showed potential to be a nutraceutical for human health because of the ability to produce astaxanthin, which is used as a coloring agent for aquaculture [20]. Moreover, astaxanthin not only helped to protect the skin against UV-induced damage, but was also used for tumor therapies and prevented neural damage associated with age-related degeneration [21,22]. *H. pluvalis* has developed into an organism that can be cultivated on an industrial scale [23]. In addition, *H. pluvalis* can also generate chlorophylls *a* and *b*, and primary carotenoid compounds namely neoxanthin, violaxanthin, zeaxanthin, lutein, and β-carotene, which suggested their great development and commercialization [21,24]. On the other hand, *Chlorella ellipsoidea* has been shown to possess bioactive substances that have various functional properties such as immunomodulatory, anti-inflammatory, and antioxidant effects [25,26]. Violaxanthin, the major component that was isolated from *C. ellipsoidae*, showed anti-inflammatory effect through inhibiting the NF-kB pathway [26]. Recent studies showed that *C. ellipsoidea* extract had significant apoptosis effect in human colon cancer cell line and suggested to have potential to prevent human cancer progression [6]. Moreover, *C. ellipsoidea* has been frequently used as a model organism in the field of genetics and the molecular biology in photosynthesis [6,27]. These observations indicated that microalgae have drawn more attention in scientific research. *Aegagropila linnaei* is a freshwater macroalga that is generally regarded as a rare and endangered species and belongs to *Cladophorales* order [28]. Velvety in appearance, these species can be excellent houseplants and are good to use in clear hanging vessels [29]. So far, the classification within the *Cladophorales* is still uncertain due to the polyphyletic nature of the large genus *Cladophora*, which results from a simple morphology with few specificity, extensive phenotypic plasticity, and both parallel and convergent evolutional character. Therefore, molecular data have contributed greatly a better understanding of *Cladophorales* evolution in recent years [30]. Unfortunately, *A. linnaei* has been used only in few molecular phylogenetic studies until now [31].

Three types of green algae were collected from a local commercial company (Available online: http://www.leadingtec.cn/). High quality genomic DNA was isolated by a modified CTAB method [32] to establish a genomic library with TrueSeq PCR-free kit and later we performed paired-end sequencing by Illumina HiSeq X Ten within 150 base pair (bp)-length unit. Paired-end deep sequencing reads were assembled by FLASH software [33] and then de novo assembled by CLCbio software (Available online: http://www.clcbio.com/) with default parameter settings (Kmer = 24, bubble size = 50). BLAST tool was applied to explore potential contigs that matched 18-5.8-28S rRNA repeats. Additional rRNA-related reads were obtained by repeatedly mapping with Geneious R9 software (Available online: http://www.geneious.com/) with 25–100 iterations. Finally, the complete 18-5.8-28S rRNA repeated unit consensus sequences were generated from the mapped reads, and were deposited to NCBI GenBank (detail information are listed in Table 1). In the three green algae tested, we found only 0.02 to 0.24% total reads were matched to 18-5.8-28S rRNA repeats. The average assembly coverage for 18-5.8-28S rRNA loci ranged from to 26 to 2736 X among the three green algal species (Table 1). These results suggested that the 18-5.8-28S rRNA copy numbers have great variation between different green algal species.

Next, we used rRNA prediction tool (Available online: http://weizhong-lab.ucsd.edu/metagenomic-analysis/server/hmm_rRNA/) and BLAST [34] to compare the gene annotation in other algal species and confirmed each identity and sequences of rRNA and ITS manually. The complete 18-5.8-28S repeats of three green algae ranged from 5785 to 6028 bps. The sequence identities of three species 18-5.8-28S rRNA repeats were confirmed by BLAST showing high identities (>99%) with previous published partial 18S rRNA sequences from the NCBI database (Figure S1). Sequence alignment of green algal rRNA repeats was generated by MAFFT [35] with default settings. High variation was detected in ITS1 and ITS2 regions (sequence identities ranged from 28.8% to 34.8% and 26.1% to 38.8%, respectively), while other regions of 18S, 5.8S and 28S (78.3% to 92.7%) were highly conserved among the three green algal species. We also used DnaSP V5 [36] to calculate the nucleotide diversity of 18-5.8-28S repeated units among three green algae. First, the nucleotide sequences were aligned by MAFFT and output as .meg file. Next, the nucleotide diversity of the aligned sequences was calculated in a sliding window with length and step size of 100 and 5 sites, respectively. Compared to rRNA regions, high nucleotide diversity (π is around 0.5–0.6) in ITS1 and ITS2 regions are detected (Figure 1). 

To validate the phylogeny of three green algae, we used MEGA6 software [37] to construct a Maximum likelihood tree (with 500 bootstrap replicates and Kimura 2-parameter model), which contains 25 species derived from Phylum Chlorophyta. *Alexandrium tamarense* [38] derived from Phylum Dinoflagellata was used as outgroup for tree rooting. The result showed that, *Chlorella ellipsoidea* is closely related to *Micractinium reisseri*; *Haematococcus pluvialis* is closely related to *Chlamydomonas* sp*.* (Figure 2). The phylogenetic relationship obtained from complete 18-5.8-28S rDNA is consistent with previous research, which used short or partial sequences from 18S or other markers [39,40]. The freshwater macroalgae, *Aegagropila linnaei*, in contrast, was shown to be phylogenetically distinct from *Chlorella ellipsoidea*, *Haematococcus pluvialis* and other green algal species tested in this study (Figure 2). In conclusion, the 18-5.8-28S rRNA repeats deduced in this study provides an important DNA data for further phylogenetic and evolutionary analysis in green algae.

Molecular-based analysis on phylogeny has started on a new page of evolutionary relationship and substituted the previous morphological-based classification. Several markers, for example, small subunit (SSU) rDNA [31], large subunit (LSU) rDNA [41], have been established to study algal phylogeny and evolution. In addition, the detail inheritance may be accomplished by developing specific protein coding genes, such as *rbcL* and *matK* in chloroplast [42,43], internal transcribed spacer (ITS) [35], and nrDNA [44]. Generally, short and ease amplified sequence, such as ITS in fungi, *rbcL* and *matK* plastid loci in plants, and mitochondrial cytochrome c oxidase subunit 1 (*cox1*) in animals, have provided a convenient tool to perform DNA barcoding [45]. Moreover, NGS technology has advanced a genomics approach to differentiate more precisely among orthologous and paralogous regions at different loci within different species. The obstacle that scientists faced was that the short sequences may not support to all branches in a phylogeny [41]. Two-locus barcoding with *rbcL* and *matK* instead of single marker analyses has improved the accuracy and resolution of phylogenetic reconstruction [46]. However, the loss of deeper branches with short markers may also reduce its resolution in the case of two closely related species with nearly identical sequence. Although LSU was able to resolve ancient eukaryotic lineage [47], the SSU was more robust when decoding deeper divergence within LSU rRNA trees [48]. While *cox1* was a suitable marker for most of animals, the slow rate of *cox1* evolution in plants may impede its scientific application [49]. The same situation is reported in some coral species which show nearly identical *cox1* sequences between closely related species [50]. In contrast, high nucleotide diversity was reported in closely species or cryptic species [51]. This situation is very common in insects who display great genomic diversity and making it difficult to design universal primers to successfully amplify target marker genes [52,53]. The advantage of NGS is that hundreds to thousands markers can be easily discovered by high coverage whole genome or even reduced genome sequencing [54]. The 18-5.8-28S rRNA repeat is a highly copied genomic unit that plays an important function during protein translation [55]. The tandemly repeated head-to-tail organization has been considered the standard for eukaryotes, which has developed into a promising approach for phylogenetic reconstruction [55]. This high-copy-number trait makes it efficient to assemble with high coverage even in low coverage whole genome sequencing. Our approach is able to bypass the restricted and time-consuming works needed to design PCR primers to amplify the complete 18-5.8-28S rRNA repeat.

Intragenomic diversity is generally low due to concerted evolution [56]. This mechanism will lead individual repeats in the multigene family to evolve in concert, resulting in the homogenization of all the repeats in an array. However, recent findings in some species, including *Haematococcus*, pointed out the intragenomic variation of rRNA may be present, which indicated that more attention to be paid since this variation might affect the species delamination [57,58,59,60]. In our study, we obtained the complete 18-5.8-28S rRNA cluster by de novo assembly, mapping and consensus sequence generation approach. The parameter setting that we used to do de novo assembly and consensus sequence generation was able to remove low quality sequencing reads/errors, sequence variation, such as SNP or potential indels. The intragenomic variation reported in other species is around the level of 0–10% [61,62,63] and we believe that this tiny variation would be removed in our current analysis pipeline. Further studies using SNP/indel calling programs, such as SAMtools/BCFtools [64] or VarScan [65] allowed us to detect more possible intragenomic variation. 

In conclusion, we provided a rapid and universal approach to deduce the complete 18-5.8-28S rRNA repeat sequences from evolutionarily diverse green algal species where the design of universal primers to amplify this locus is not possible. In addition, the complete 18-5.8-28S rRNA repeat unit sequence provides a good nuclear marker for phylogenetic and evolutionary analysis for green algae.

## Figures and Tables

**Figure 1:**
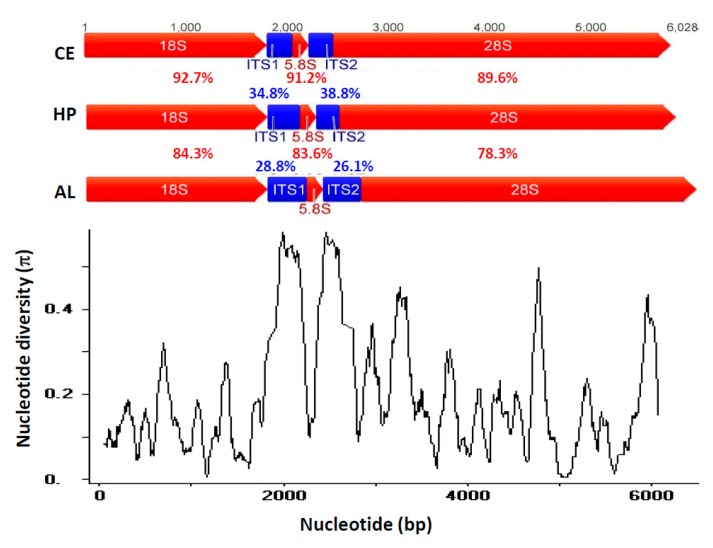
The complete 18-5.8-28S rRNA repeat unit of three green algae, *Chlorella ellipsoidea*, *Haematococcus pluvialis* and *Aegagropila linnaei* (Phylum Chlorophyta). The 18S, 5.8S and 28S rRNA genes are labeled in red, ITS1 and ITS2 sequences are labeled in blue. The nucleotide sequence identities are also highlighted for comparison. Lower panel shows the sliding window to compare the nucleotide diversity of 18-5.8-28S rDNA repeat unit assembly among three green algal species.

**Figure 2:**
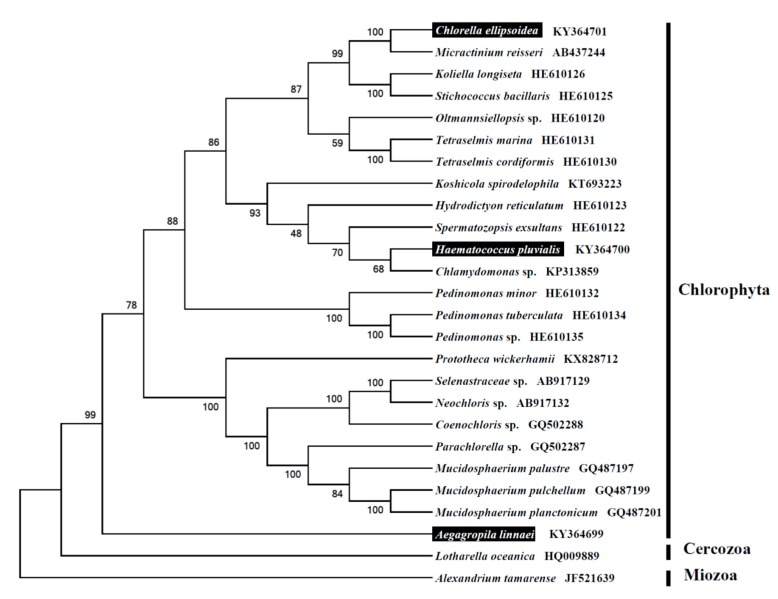
Molecular phylogeny of three green algae and related species in Phylum Chlorophyta based on complete 18-5.8-28S rRNA repeat units. The complete or partial 18-5.8-28S rRNA repeat sequences were downloaded from GenBank to construct a phylogenic tree by the Maximum likelihood method with 500 bootstrap replicates. Three targeted green algal *Chlorella ellipsoidea*, *Haematococcus pluvialis* and *Aegagropila linnaei*, are highlighted in black.

**Table 1:** Summary of three algae species tested in this study.

Species	*Chlorella ellipsoidea* (CE)	*Haematococcus pluvialis* (HP)	*Aegagropila linnaei* (AL)
Total reads	67,016,212	68,697,604	7,502,824
18-5.8-28S reads	159,445	74,748	1648
18-5.8-28S reads % *	0.24	0.11	0.02
Coverage (fold)	2736	825	26
Sequence (bp)	5785	5817	6028
NCBI accession number	KY364701	KY364700	KY364699

* This is defined as (18-5.8-28S reads/Total reads) × 100.

## Supplementary Materials

The following are available online at www.mdpi.com/1422-0067/18/11/2341/s1.
