# Supplementary material for: A Simple Method to Decode the Complete 18-5.8-28S rRNA Repeated Units of Green Algae by Genome Skimming
Source: Int J Mol Sci. 2017 Nov 6;18(11):2341. doi: 10.3390/ijms18112341 (PMC5713310; doi:10.3390/ijms18112341)
Supplement: Supplementary file 1 [file ijms-18-02341-s001.pdf]

Figure S1_BLAST hints for three green algae 18-5.8-28S rRNA

BLAST hint for *Haematococcus pluvialis* 18-5.8-28S rRNA sequence

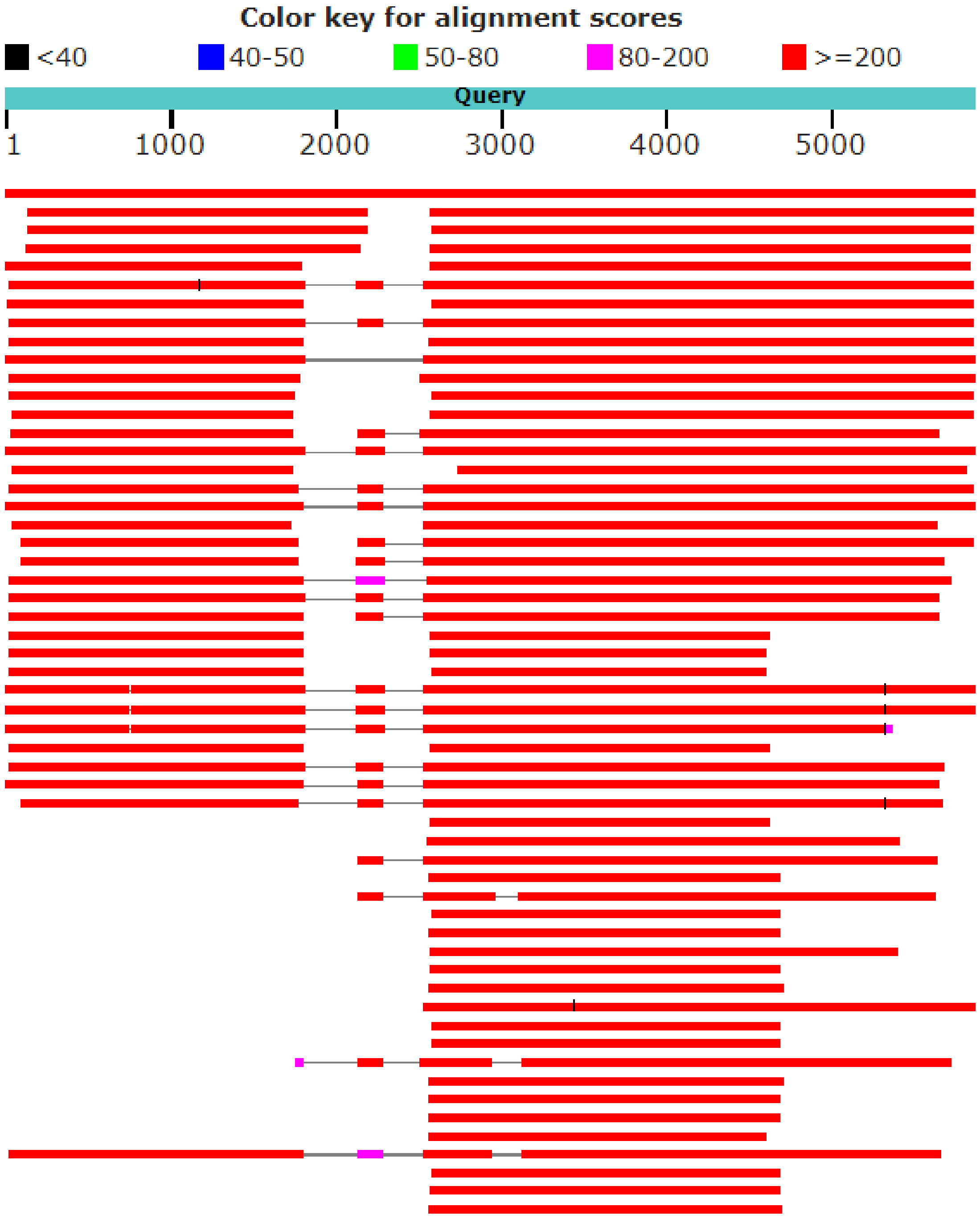

Sequence alignment result for *Haematococcus pluvialis* 18-5.8-28S rRNA sequence

Haematococcus pluvialis strain KMMCC 1354 18S ribosomal RNA gene, partial sequence

Sequence ID: JQ315539.1 **Length:** 2040 **Number of Matches:** 1

Range 1: 1 to 2040 GenBank Graphics ▼ Next Match ▲ Previous Match

Score	Expect	Identities	Gaps	Strand
3738 bits(2024)	0.0	2036/2041(99%)	3/2041(0%)	Plus/Plus

```
Query  142   ACCG-TAGTAATTCTAGAGCTAATACGTGCG-TATATCCCGACTTCTGGAAGGGACGTAT  199
             |||| |||| ||||||||||||||||||||| ||||||||||||||||||||||||||||
Sbjct  1     ACCGCTAGT-ATTCTAGAGCTAATACGTGCGCTATATCCCGACTTCTGGAAGGGACGTAT  59

Query  200   TTATTAGATAAAAGGCCAGCCGGGCTTGCCCGACCTATGGCGAATCATGATAACTTCACG  259
             ||||||||||||||||||||||||||||||||||||||||||||||||||||||||||||
Sbjct  60    TTATTAGATAAAAGGCCAGCCGGGCTTGCCCGACCTATGGCGAATCATGATAACTTCACG  119

Query  260   AATCGCACGGCCTTGCGCCGGCGATGTTTCATTCAAATTTCTGCCCTATCAACTTTCGAT  319
             ||||||||||||||||||||||||||||||||||||||||||||||||||||||||||||
Sbjct  120   AATCGCACGGCCTTGCGCCGGCGATGTTTCATTCAAATTTCTGCCCTATCAACTTTCGAT  179

Query  320   GGTAGGATAGAGGCCTACCATGGTGGTAACGGGTGACGGAGGATTAGGGTTCGATTCCGG  379
             ||||||||||||||||||||||||||||||||||||||||||||||||||||||||||||
Sbjct  180   GGTAGGATAGAGGCCTACCATGGTGGTAACGGGTGACGGAGGATTAGGGTTCGATTCCGG  239

Query  380   AGAGGGAGCCTGAGAAACGGCTACCACATCCAAGGAAGGCAGCAGGCGCGCAAATTACCC  439
             ||||||||||||||||||||||||||||||||||||||||||||||||||||||||||||
Sbjct  240   AGAGGGAGCCTGAGAAACGGCTACCACATCCAAGGAAGGCAGCAGGCGCGCAAATTACCC  299

Query  440   AATCCCGACACGGGGAGGTAGTGACAATAAATAACAATACCGGGCATCAATGTCTGGTAA  499
             ||||||||||||||||||||||||||||||||||||||||||||||||||||||||||||
Sbjct  300   AATCCCGACACGGGGAGGTAGTGACAATAAATAACAATACCGGGCATCAATGTCTGGTAA  359

Query  500   TTGGAATGAGAACAATTTAAATCCCTTAACGAGTATCCATTGGAGGGCAAGTCTGGTGCC  559
             ||||||||||||||||||||||||||||||||||||||||||||||||||||||||||||
Sbjct  360   TTGGAATGAGAACAATTTAAATCCCTTAACGAGTATCCATTGGAGGGCAAGTCTGGTGCC  419

Query  560   AGCAGCCGCGGTAATTCCAGCTCCAATAGCGTATATTTAAGTTGTTGCAGTTAAAAAGCT  619
             ||||||||||||||||||||||||||||||||||||||||||||||||||||||||||||
Sbjct  420   AGCAGCCGCGGTAATTCCAGCTCCAATAGCGTATATTTAAGTTGTTGCAGTTAAAAAGCT  479

Query  620   CGTAGTTGGATTTCGGGTGGGTTCCAGCGGTCTGCCTCTGGTATGTACTGCTGTGGCCTA  679
             ||||||||||||||||||||||||||||||||||||||||||||||||||||||||||||
Sbjct  480   CGTAGTTGGATTTCGGGTGGGTTCCAGCGGTCTGCCTCTGGTATGTACTGCTGTGGCCTA  539

Query  680   CCTTTCTGCCGGGGACGTGTTCCTGGGCTTCATTGTCCGGGACTCGAATTCGGCGAGGAT  739
             ||||||||||||||||||||||||||||||||||||||||||||||||||||||||||||
Sbjct  540   CCTTTCTGCCGGGGACGTGTTCCTGGGCTTCATTGTCCGGGACTCGAATTCGGCGAGGAT  599

Query  740   ACTTTGAGTAAAACAGCGTGTTCAAAGCAAGCCTACGCTCTGAATGCATTAGCATGGAAT  799
             ||||||||||||||||||||||||||||||||||||||||||||||||||||||||||||
Sbjct  600   ACTTTGAGTAAAACAGCGTGTTCAAAGCAAGCCTACGCTCTGAATGCATTAGCATGGAAT  659

Query  800   ATCACGATAGGACTCTGGCCTATCTTGTTGGTCTGTAGGACCGGAGTAATGATTAAGAGG  859
             ||||||||||||||||||||||||||||||||||||||||||||||||||||||||||||
Sbjct  660   ATCACGATAGGACTCTGGCCTATCTTGTTGGTCTGTAGGACCGGAGTAATGATTAAGAGG  719

Query  860   GACAGTCGGGGGCATTCGTATTTCATTGTCAGAGGTGAAATTCTTGGATTTATGAAAGAC  919
             ||||||||||||||||||||||||||||||||||||||||||||||||||||||||||||
Sbjct  720   GACAGTCGGGGGCATTCGTATTTCATTGTCAGAGGTGAAATTCTTGGATTTATGAAAGAC  779
```

BLAST hint for *Aegagropila linnaei* 18-5.8-28S rRNA sequence

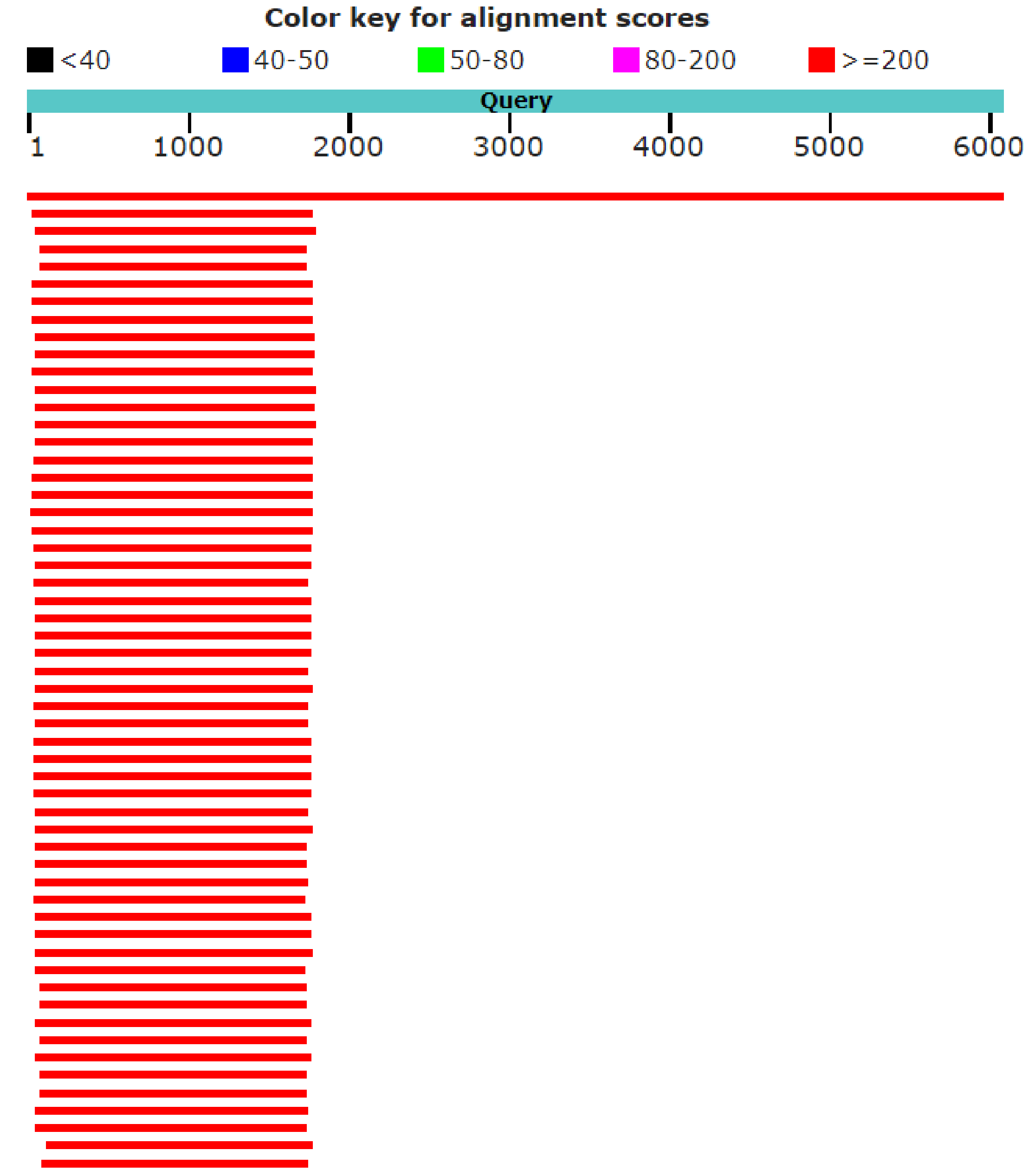

Sequence alignment result for *Aegagropila linnaei* 18-5.8-28S rRNA sequence

Aegagropila linnaei gene for 18S ribosomal RNA, partial sequence, Lake Akan

Sequence ID: AB062698.1 **Length:** 1738 **Number of Matches:** 1

▶ See 3 more title(s)

Range 1: 1 to 1738 GenBank Graphics ▼ Next Match ▲ Previous Match

Score	Expect	Identities	Gaps	Strand
3205 bits(1735)	0.0	1738/1739(99%)	1/1739(0%)	Plus/Plus

```
Query  20   TCGTGATATGCTTGTCTCAAAGATTAAGCCATGCATGTGTAGGTATAACCGATTATACCG  79
            ||||||||||||||||||||||||||||||||||||||||||||||||||||||||||||
Sbjct  1    TCGTGATATGCTTGTCTCAAAGATTAAGCCATGCATGTGTAGGTATAACCGATTATACCG  60

Query  80   GAAAACTGCGAATGGCTCGGTAAATCAGTTATAGTTTATTTGATAGTGCTCACTACTCGG  139
            ||||||||||||||||||||||||||||||||||||||||||||||||||||||||||||
Sbjct  61   GAAAACTGCGAATGGCTCGGTAAATCAGTTATAGTTTATTTGATAGTGCTCACTACTCGG  120

Query  140  ATAACCGTAGTAACGCTAGAGCTAATACGTGCGTAAATCCCGACTCTAGGAAGGGACGTA  199
            ||||||||||||||||||||||||||||||||||||||||||||||||||||||||||||
Sbjct  121  ATAACCGTAGTAACGCTAGAGCTAATACGTGCGTAAATCCCGACTCTAGGAAGGGACGTA  180

Query  200  TTTATTAGATAAAAGGCCGACCGGGTTTCCCGATCTGCGCTGAATCATGGTAACTTCACG  259
            ||||||||||||||||||||||||||||||||||||||||||||||||||||||||||||
Sbjct  181  TTTATTAGATAAAAGGCCGACCGGGTTTCCCGATCTGCGCTGAATCATGGTAACTTCACG  240

Query  260  GATTGCATGGCCTTCGTGCCAGCGACGTGTCATTCAAGTTTCTGCCCCATCATGGTTTCG  319
            ||||||||||||||||||||||||||||||||||||||||||||||||||||||||||||
Sbjct  241  GATTGCATGGCCTTCGTGCCAGCGACGTGTCATTCAAGTTTCTGCCCCATCATGGTTTCG  300

Query  320  ACTGTAATGTATTGGATTACAGTGCCGATAACGGGTAGCGGAGGATTAGGGTTCGATTCC  379
            ||||||||||||||||||||||||||||||||||||||||||||||||||||||||||||
Sbjct  301  ACTGTAATGTATTGGATTACAGTGCCGATAACGGGTAGCGGAGGATTAGGGTTCGATTCC  360

Query  380  GGAGAGGGCGCCTGAGAAATGGCGACCACATCCAAGGAAGGCAGCAGGCGCGCAAATTAC  439
            ||||||||||||||||||||||||||||||||||||||||||||||||||||||||||||
Sbjct  361  GGAGAGGGCGCCTGAGAAATGGCGACCACATCCAAGGAAGGCAGCAGGCGCGCAAATTAC  420

Query  440  CCAATCCCAACTCAGGGAGGTAGTGACAAGAAATAACAATGCGGAGCCATAGGTTCTGCA  499
            ||||||||||||||||||||||||||||||||||||||||||||||||||||||||||||
Sbjct  421  CCAATCCCAACTCAGGGAGGTAGTGACAAGAAATAACAATGCGGAGCCATAGGTTCTGCA  480

Query  500  ATTGGAATGAGTACAATTTAAACCACTTAACGAGTACCTATTGGAGGGCAAGTCTGGTGC  559
            ||||||||||||||||||||||||||||||||||||||||||||||||||||||||||||
Sbjct  481  ATTGGAATGAGTACAATTTAAACCACTTAACGAGTACCTATTGGAGGGCAAGTCTGGTGC  540

Query  560  CAGCAGCCGCGGTAATTCCAGCTCCAATAGCGTATATGTAAGTTGTTGCGATTAAAAAGC  619
            ||||||||||||||||||||||||||||||||||||||||||||||||||||||||||||
Sbjct  541  CAGCAGCCGCGGTAATTCCAGCTCCAATAGCGTATATGTAAGTTGTTGCGATTAAAAAGC  600

Query  620  CCGTAGCTGAACCTCGGGCAGCCCGGACGGTCCGCCTTACGGTGAGTACTGTCCTGGGCT  679
            ||||||||||||||||||||||||||||||||||||||||||||||||||||||||||||
Sbjct  601  CCGTAGCTGAACCTCGGGCAGCCCGGACGGTCCGCCTTACGGTGAGTACTGTCCTGGGCT  660
```

BLAST hint for *Chlorella ellipsoidea* 18-5.8-28S rRNA sequence

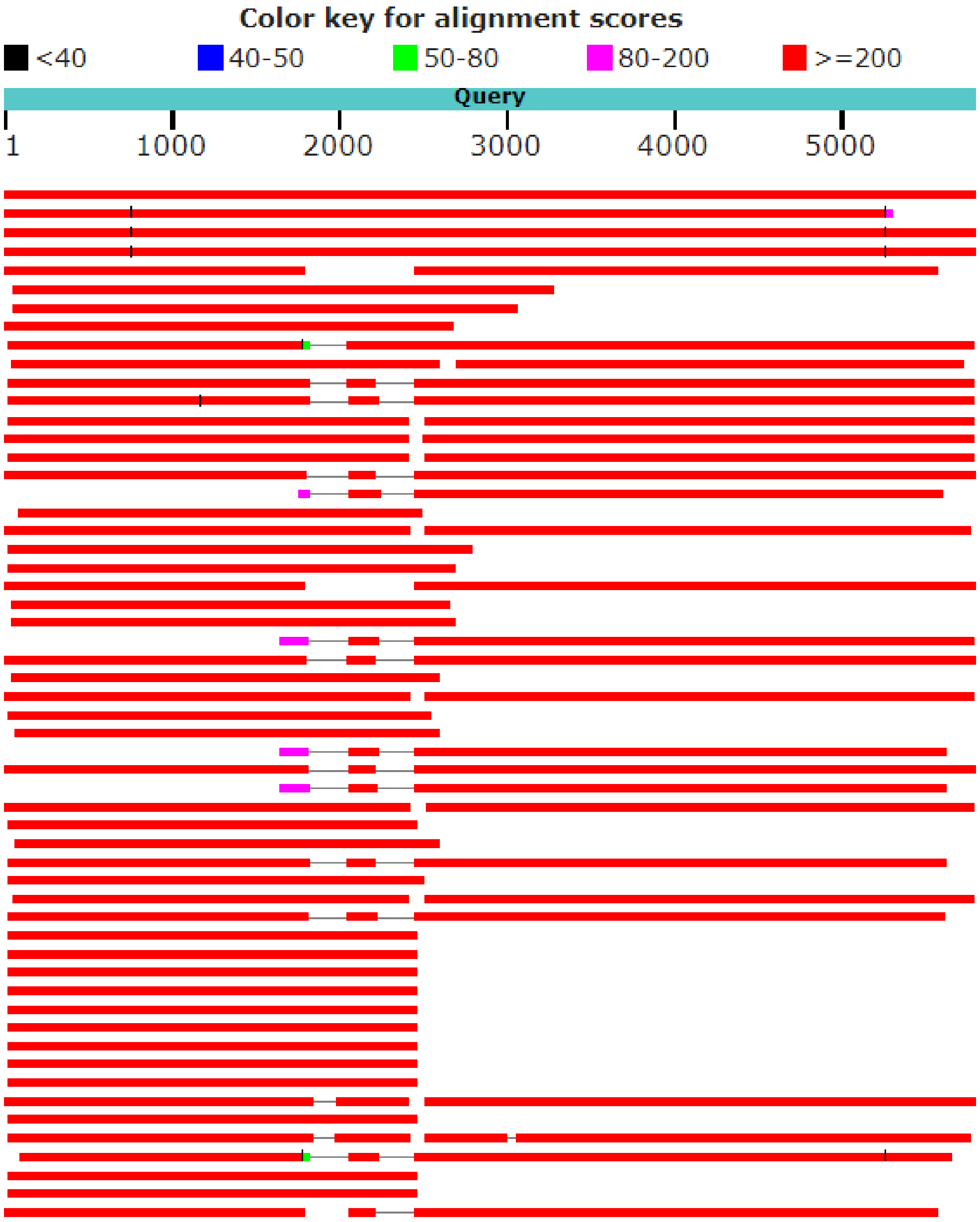

Sequence alignment result for *Chlorella ellipsoidea* 18-5.8-28S rRNA sequence

Chlorella sorokiniana 18S rRNA gene, strain Prag A14

Sequence ID: X74001.1 **Length:** 1797 **Number of Matches:** 1

Range 1: 2 to 1796 GenBank Graphics ▼ Next Match ▲ Previous Match

Score	Expect	Identities	Gaps	Strand
3299 bits(1786)	0.0	1792/1795(99%)	0/1795(0%)	Plus/Plus

```
Query  1    ACCTGGTTGATCCTGCCAGTAGTCATATGCTTGTCTCAAAGATTAAGCCATGCATGTCTA  60
            ||||||||||||||||||||||||||||||||||||||||||||||||||||||||||||
Sbjct  2    ACCTGGTTGATCCTGCCAGTAGTCATATGCTTGTCTCAAAGATTAAGCCATGCATGTCTA  61

Query  61   AGTATAAACTGCTTTATACTGTGAAACTGCGAATGGCTCATTAAATCAGTTATAGTTTAT  120
            ||||||||||||||||||||||||||||||||||||||||||||||||||||||||||||
Sbjct  62   AGTATAAACTGCTTTATACTGTGAAACTGCGAATGGCTCATTAAATCAGTTATAGTTTAT  121

Query  121  TTGATGGTACCTACTACTCGGATACCCGTAGTAAATCTAGAGCTAATACGTGCGTAAATC  180
            ||||||||||||||||||||||||||||||||||||||||||||||||||||||||||||
Sbjct  122  TTGATGGTACCTACTACTCGGATACCCGTAGTAAATCTAGAGCTAATACGTGCGTAAATC  181

Query  181  CCGACTTCTGGAAGGGACGTATTTATTAGATAAAAGGCCGACCGGGCTCTGCCCGACTCG  240
            ||||||||||||||||||||||||||||||||||||||||||||||||||||||||||||
Sbjct  182  CCGACTTCTGGAAGGGACGTATTTATTAGATAAAAGGCCGACCGGGCTCTGCCCGACTCG  241

Query  241  CGGTGAATCATGATAACTTCACGAATCGCATGGCCTCGTGCCGGCGATGTTTCATTCAAA  300
            ||||||||||||||||||||||||||||||||||||||||||||||||||||||||||||
Sbjct  242  CGGTGAATCATGATAACTTCACGAATCGCATGGCCTCGTGCCGGCGATGTTTCATTCAAA  301

Query  301  TTTCTGCCCTATCAACTTTCGATGGTAGGATAGAGGCCTACCATGGTGGTAACGGGTGAC  360
            ||||||||||||||||||||||||||||||||||||||||||||||||||||||||||||
Sbjct  302  TTTCTGCCCTATCAACTTTCGATGGTAGGATAGAGGCCTACCATGGTGGTAACGGGTGAC  361

Query  361  GGAGGATTAGGGTTCGATTCCGGAGAGGGAGCCTGAGAAACGGCTACCACATCCAAGGAA  420
            ||||||||||||||||||||||||||||||||||||||||||||||||||||||||||||
Sbjct  362  GGAGGATTAGGGTTCGATTCCGGAGAGGGAGCCTGAGAAACGGCTACCACATCCAAGGAA  421

Query  421  GGCAGCAGGCGCGCAAATTACCCAATCCTGACACAGGGAGGTAGTGACAATAAATAACAA  480
            ||||||||||||||||||||||||||||||||||||||||||||||||||||||||||||
Sbjct  422  GGCAGCAGGCGCGCAAATTACCCAATCCTGACACAGGGAGGTAGTGACAATAAATAACAA  481

Query  481  TACTGGGCCTTTTCAGGTCTGGTAATTGGAATGAGTACAATCTAAACCCCTTAACGAGGA  540
            ||||||||||||||||||||||||||||||||||||||||||||||||||||||||||||
Sbjct  482  TACTGGGCCTTTTCAGGTCTGGTAATTGGAATGAGTACAATCTAAACCCCTTAACGAGGA  541

Query  541  TCAATTGGAGGGCAAGTCTGGTGCCAGCAGCCGCGGTAATTCCAGCTCCAATAGCGTATA  600
            ||||||||||||||||||||||||||||||||||||||||||||||||||||||||||||
Sbjct  542  TCAATTGGAGGGCAAGTCTGGTGCCAGCAGCCGCGGTAATTCCAGCTCCAATAGCGTATA  601

Query  601  TTTAAGTTGCTGCAGTTAAAAAGCTCGTAGTTGGATTTCGGGTGGGGCCTGCCGGTCCGC  660
            ||||||||||||||||||||||||||||||||||||||||||||||||||||||||||||
Sbjct  602  TTTAAGTTGCTGCAGTTAAAAAGCTCGTAGTTGGATTTCGGGTGGGGCCTGCCGGTCCGC  661

Query  661  CGTTTCGGTGTGCACTGGCAGGGCCCACCTTGTTGCCGGGGACGGGCTCCTGGGCTTCAC  720
            ||||||||||||||||||||||||||||||||||||||||||||||||||||||||||||
Sbjct  662  CGTTTCGGTGTGCACTGGCAGGGCCCACCTTGTTGCCGGGGACGGGCTCCTGGGCTTCAC  721
```
